# “They Don’t Really Consider Us Essential, But We Are”: a Qualitative Investigation of Vaccine Acceptance and Perceived Workplace Safety Among Black Transit Workers During the COVID-19 Pandemic

**DOI:** 10.1007/s40615-023-01606-5

**Published:** 2023-05-23

**Authors:** Khadijah Ameen, Denise T. St Jean, Chioma Woko

**Affiliations:** 1 Department of Health Promotion and Behavior, Georgia State University School of Public Health, 140 Decatur St SE, Atlanta, GA 30303 USA; 2 Department of Epidemiology, Gillings School of Global Public Health, University of North Carolina at Chapel Hill, Chapel Hill, NC USA; 3 Annenberg School for Communication, University of Pennsylvania, Philadelphia, PA USA

**Keywords:** COVID-19, Occupational health, Racism, Vaccine hesitancy, Health equity

## Abstract

Black Americans face a higher risk of coronavirus disease 2019 (COVID-19) morbidity and mortality due to adverse social determinants of health, including their overrepresentation in the frontline workforce. Despite these inequities, increasing vaccine acceptance among this subpopulation has been challenging. We conducted semi-structured qualitative focus groups with Black public transit workers living in the USA to explore behavioral intentions regarding COVID-19 vaccine uptake, occupational health challenges, and the perceived impact of racism on workplace health and safety during the pandemic. A thematic analysis approach was used to analyze the final transcripts. We completed three focus groups (*n*=10 participants) in October and November of 2021. Enabling factors for vaccination included opportunities for vaccination in the workplace, flexible hours of operation, and walk-in vaccine clinics. Disabling factors included excessive wait times. Some participants also cited lack of cleanliness, inconsistent enforcement of COVID-19 safety protocols, and unclear workplace policies regarding sick and hazard pay as major safety barriers. Perceptions regarding the role of racism in their experiences with COVID-19 as transit workers were mixed. Though occupational health and safety concerns were high, there are opportunities for transit agencies and government officials to improve both vaccine uptake and working conditions for Black transit workers.

## Introduction

The coronavirus disease 2019 (COVID-19) pandemic has exacerbated existing health inequities, particularly among workers who have been deemed “essential” [1, 2]. Among frontline workers, public transit workers have served a critical role in the functioning of society throughout the COVID-19 pandemic. Yet, despite their central role, the health and safety of this group has not been prioritized in the USA [3]. At the height of the pandemic, COVID-19 prevalence among transit workers was as high as 25% in some states [4]. One study conducted by the California Department of Public Health found that cumulative COVID-19 outbreak incidence was 5.2 times higher and mortality 1.8 times higher in the bus and urban transit industry compared to all industries in California [5].

Gaps in COVID-19 vaccine uptake have been observed among Black Americans throughout the duration of the pandemic [6]. COVID-19 vaccine hesitancy in the USA demonstrated pronounced disparities across racial lines, particularly at the beginning of vaccine rollout [7–9]. Largely influenced by medical racism and historical untrustworthiness of scientific institutions [10], Black Americans were slower to accept the newly approved COVID-19 vaccines at the start of the pandemic [11, 12]. Lower vaccination intention among this population was concerning in the public health community due to the disproportionate impact the pandemic has had on Black populations [13]. Black Americans remain 2.3 times more likely to be hospitalized from COVID-19, and 1.7 times more likely to die from COVID-19 than White non-Hispanic Americans [14]. These differences have been explained by determinants of health that increase race-based vulnerabilities to COVID-19, such as racism, poverty, barriers to health care access, overcrowded living conditions, environmental hazards, and overrepresentation in the frontline workforce [15–17]

Black workers’ disproportionate representation on the public transit frontline increases their social vulnerability to SARS-CoV-2 exposure [18]. While Black workers represent only 12% of the employed workforce, 26% of public transit workers identify as Black [18]. In urban centers like New York City, Black workers represent an even larger percentage of the public transit workforce, with 46% of transit workers identifying as Black or African American [19, 20]. Systemic racism in occupational health policy dating back to chattel slavery has led to sub-standard working conditions, lower wages, limited benefits, and an overemphasis of productivity over safety for Black workers, increasing risk of adverse health outcomes [21, 22,23, 24]. These factors make it important to understand and address facilitators and barriers to vaccine uptake that might exist within this group.

The objective of this exploratory qualitative study was to learn about the concerns, barriers, and facilitators of COVID-19 vaccine acceptance among Black transit workers in order to inform occupational health policy and health communication strategies during future pandemics. Through the use of semi-structured qualitative focus groups with Black public transit workers living in the USA, we explored three domains related to their first-hand experiences with COVID-19 in the workplace: (1) behavioral intentions around COVID-19 vaccine uptake, and the enabling and disabling factors influencing those intentions; (2) workplace health and safety meaning-making, including occupational health challenges experienced during the pandemic and suggestions for advancing workplace safety; and (3) perceived impact of racism on workplace health and safety experiences during the pandemic.

## Methods

### Recruitment Strategy

We used a combined criterion-based and snowball purposeful sampling strategy. Transit workers were invited to participate in the focus groups through targeted outreach to local chapters of the Amalgamated Transit Union, the largest labor union representing transit and allied workers in the USA and Canada. Additional transit workers were recruited through snowball sampling from focus group participants. Participation in this study was limited to public transit workers in the USA, as defined by 53-3052 Bus Drivers, Transit and Intercity, Standard Occupational Classification, who self-identified as Black or African American, and were 18 years of age or older and able to provide informed consent. Study participation also required access to a device that allowed for video conferencing, as well as access to broadband internet. Transit workers who did not meet the occupational, age, and racial identity requirement, or who lacked access to the technological equipment required to participate in the focus groups, were excluded from the study. Additionally, those who had previously enrolled in a COVID-19 vaccine trial were excluded from the study. The study was approved by the Johns Hopkins Bloomberg School of Public Health Institutional Review Board (IRB #: 15601) and informed consent was obtained from all study participants.

### Focus Group Data Collection Procedures

We conducted a total of three virtual focus groups with three to four research participants in each group (*n*=10). The focus groups lasted 60 to 90 min each. Focus groups were conducted between October and November of 2021. An open-ended, semi-structured focus group discussion guide was developed to explore participants’ behavioral intentions regarding the COVID-19 vaccine. The discussion guide was developed using the Theory of Reasoned Action, which explains that behavioral adoption is influenced by behavioral, normative, and self-efficacy beliefs [25]. Participants were also probed on vaccine hesitancy-specific constructs delineated in the World Health Organization’s Increasing Vaccination Model [26], their perceptions of their workplace safety during the COVID-19 pandemic as Black transit workers, and their proposed recommendations for improving workplace health and safety.

After obtaining verbal informed consent, focus groups were held virtually using the Zoom video-conferencing platform. Principal investigators of the study were present for and facilitated all focus group discussions. Each focus group was audio-recorded within Zoom and transcribed by Rev transcription service. Immediately following the discussion, participants were asked to complete a brief Qualtrics exit survey to collect demographic, COVID-19 vaccination, and occupational health information. Participants received $150 via electronic Visa gift card or Venmo for completing the focus group and the exit survey.

### Focus Group Data Analysis

Transcripts from Rev transcription service were reviewed and cleaned by a primary qualitative coder in order to remove any identifiers and correct any transcription errors. Microsoft Excel was then utilized to code the content derived from the final transcripts. A thematic analysis approach was used to code data from the final transcripts. Initial codes were first created inductively through open coding to identify discrete concepts that arose from the three focus group sessions. Discrete codes were then reduced into categories based on overlapping concepts across the focus groups. Finally, categories were further collated and reorganized into themes in order to formulate relationships between categories. Themes were pre-defined and reflective of the Theory of Reasoned Action constructs within the focus group discussion guide*.* During the coding process, an intercoder reliability exercise was conducted with two additional coders to reduce bias and gain consensus between coders (final Cohen’s Kappa = 0.90). Pseudonyms were used for all quotes referenced in the “Results” section to protect the identities of participants.

### Participant Characteristics

We completed a total of three focus groups that included 10 participants. All participants completed the Qualtrics exit survey. Results from the exit survey are included in Table 1. Ninety percent of participants were from New York and all participants worked full-time as transit workers. The median participant age was 48 years (interquartile range, 36–52 years), and 60% of participants were men. Sixty percent of participants reported having previously tested positive for COVID-19. Of those, 83% (*n*=5) believed that their infection stemmed from workplace exposure.

## Results

### Personal Impact of COVID-19 Pandemic

*One of our members passed away from COVID…. less than two weeks ago. And the numbers are always climbing in the transit family. - Matthew*

The COVID-19 pandemic directly impacted all focus group participants in some way. Several participants shared that they had personally contracted COVID-19 and had to take time off work to recover. For others, family members and friends contracted COVID-19 and became ill from the virus. Others had witnessed mass COVID-19 infections and deaths among their transit colleagues. One participant stated that over 100 workers at their transit authority had died from COVID-19 since the start of the pandemic. The high exposure nature of the job was cited as a driver of high COVID-19 infection rates among employees.

### Personal Protection Strategies Against COVID-19

*At the beginning, we was told not to wear the PPE, to not alarm the people outside of transit. – Malika*

Participants described engaging in various strategies to protect themselves and those around them like family, friends, coworkers, and passengers from contracting COVID-19. Such strategies included masking, hand washing and hand sanitizing, and social distancing when possible. Participants who were not vaccinated stated they were engaging in alternative protective strategies to mitigate contracting and spreading COVID-19, such as wearing personal protective equipment (PPE), social distancing, and using natural remedies like vitamins. Several participants stated they were uncertain about what personal protective strategies to engage in due to factors like changing workplace protocols and mixed health communications messaging. One participant mentioned that they were initially advised by their management not to wear PPE in order to curb public alarm. Another participant shared that they felt there was little that transit workers could do to prevent exposure due to the nature of the job.

### Knowledge About the COVID-19 Vaccine

*A big skepticism for me and in our transit system is the commercials, the Hollywood stars, the way they're pushing it. It makes me feel uncomfortable. - Marquis*

Several participants expressed personal skepticism and skepticism among family, friends, and colleagues about the COVID-19 vaccine based on information they had received. Participants shared that they had heard that the COVID-19 vaccine development process was rushed, that the COVID-19 vaccine caused serious side effects, and that vaccinated individuals were still succumbing to breakthrough infections. More than one participant shared that they felt that health communications to get vaccinated against COVID-19 were being “pushed” at them. Several participants shared that the sources of COVID-19 vaccine information (e.g., the media, federal government, and peers) were presenting conflicting and incomplete information, leading to difficulty deciphering factual information from misinformation and disinformation. Despite skepticism and confusion, some participants stated that they had heard positive information about the COVID-19 vaccine, like that it would prevent severe COVID-related illness and death. Ultimately, several participants stated that they had to do their own research to inform their decision-making regarding whether or not to get vaccinated against COVID-19.

### Perceived Advantages and Disadvantages of the COVID-19 Vaccine

Perceived advantages of the COVID-19 vaccine shared by focus group participants included physical benefits like reduction in severity of illness and faster recovery if infected. One participant stated that given the high exposure nature of working in transit, the COVID-19 vaccine could provide an added layer of protection against the virus. Social benefits shared included protecting others against COVID-19 infection, being able to continue everyday social activities, and being relieved of the social pressure to get vaccinated. Community-level perceived advantages included beliefs that high COVID-19 vaccination rates could contribute to herd immunity and limit the emergence of new variants.

Perceived disadvantages of the COVID-19 vaccine included side effects from the vaccine such as fever-like symptoms and potential severe complications post-vaccination. A couple of participants stated that it was too early to know the true impact of the COVID-19 vaccine and what it does to the body. One participant shared that they were skeptical about COVID-19 booster shots and the need to regularly get revaccinated against COVID-19. Another major disadvantage stated was the potential for breakthrough infections and death despite being vaccinated.

### Normative Beliefs About COVID-19 Vaccination

*Well, I work with a lot of people who are actually pushing the vaccination, and I don't have a problem with that. My motto is I believe in let it be your choice. – Shauna*

Several focus group participants shared that individuals in their interpersonal circle such as friends, family, coworkers, and transit leadership were encouraging them to get vaccinated against COVID-19. Some participants framed this support as “pressure” and “pushing,” which was met with mixed feelings by those participants. Other participants shared examples of vaccine disapproval from their social circles. For example, one participant stated that vaccine dissenters in their social group were more vocal than vaccine supporters. Another stated that despite social pressures, they would have to be forced by law or fined in order to get vaccinated against COVID-19. Regardless of their ultimate decision to get or not get vaccinated, an overwhelming number of participants expressed the importance of personal choice in the vaccine decision-making process.

### Enabling and Disabling Conditions to COVID-19 Vaccination

*Well one [facilitating factor] is my job. Before you had to make an appointment, but now you could just walk in. The job made it easier. You could just go in, on the free time, and go get vaccinated. So it wasn't that much of a wait for my job. – Aliyah*

Participants shared a variety of enabling and disabling conditions for getting vaccinated against COVID-19 in their workplaces and communities. One of the major enabling conditions shared by participants was being able to get vaccinated through their employer. In community settings, enabling conditions included simple processes for booking appointments, flexible hours of operation, and walk-in opportunities. Regarding disabling factors, one participant shared that while it was easy for her to get vaccinated at her workplace, it was difficult for her family member to get vaccinated in the community due to long wait times. Another participant shared that they did not believe accessibility was an issue in regard to transit workers getting vaccinated for COVID-19. Rather, they believed that personal beliefs about the vaccine were the leading factor in whether or not an individual worker got vaccinated.

One potential enabling condition that was not present but could have promoted COVID-19 vaccine uptake was vaccine mandates. Several participants shared that their employer did not mandate employees to get vaccinated for COVID-19. As an alternative, their workplaces encouraged vaccinations during staff meetings and through signage. One participant shared that for new hires, their workplace asked about COVID-19 vaccination status. Another participant shared that a vaccine mandate by their employer would be the only reason they would get vaccinated against COVID-19.

### Workplace Safety Mean-Making

*One of the main things in the transit world is safety before everything else. Safety before schedule. But that schedule comes in right after that. - Malika*

Workplace safety was cited as a top priority by almost all focus group participants in the face of the COVID-19 pandemic. When asked what workplace safety meant to them as transit workers, participants shared a variety of key components such as on-site free PPE provided to workers and passengers, and the ability to socially distance on trains and buses, in crew and break rooms, and when docking.

*The first thing, it has to be clean. I don’t care how many masks they wear. If they’re not cleaning down everything like they’re supposed to, then we’re not going to be safe. And for us at transit, that’s our number one priority. - Jabril*

Several participants shared that cleanliness was a mandatory feature of safety. For those participants, cleanliness required routine and thorough cleaning practices on the buses and trains and around the surrounding depots. Additional important aspects of safety that were mentioned included consistent and transparent enforcement of COVID-19 safety protocols at transit worksites, as well as the ability to take off work when sick.

### Barriers to Workplace Safety

*As far as the bathrooms, the air quality, simple things that normal people at regular jobs would get, we’re deprived of it. - Matthew*

Participants shared several barriers to workplace safety in the transit community since the COVID-19 pandemic began. One major barrier to workplace safety expressed was lack of cleanliness. Several participants shared that transit worksites were dirty due to lack of regular and thorough cleaning services. In the absence of reliable cleaning services, several participants stated that they had to clean their own workstations. Air quality was also cited as a major barrier to workplace safety due to inability to social distance, lack of clean air circulation, and potential exposure to carcinogens. This was particularly true for focus group participants who worked at underground stations.

*All they care about is moving passengers, getting all their money from the fares and so forth. They could care less about us. – Sasha*

In total, 90% of research participants indicated that their employers were not taking the appropriate actions to protect their health and safety. Participants shared that COVID-19 mitigation was not a top priority for transit leadership and that they found leadership to be reactive rather than proactive in the fight against COVID-19. Specific leadership failures that participants cited included poor dissemination and constantly changing COVID-19 workplace safety protocols and inadequate enforcement of such protocols. Participants stated that this had led to a range of issues with passengers, from passengers not wearing masks to passengers physically and verbally assaulting transit workers when asked to adhere to safety protocols. In addition to inconsistent and dwindling enforcement of COVID-19 safety protocols by leadership, participants shared that lack of clear workplace policies regarding continuity of pay if sick with COVID-19 was a major safety barrier. One participant shared that because transit workers live paycheck to paycheck, it is very difficult for them to afford missing work if they needed to quarantine.

*We fought for everything, from when they put out a bulletin soon as COVID hit telling us we were not supposed to wear masks, to fighting for masks, fighting to get tested, fighting for PPE, fighting to get the white line moved behind on the back on the bus. We fought for everything with transit. – Jamil*

In the face of substantial safety challenges that participants experienced at their workplaces, participants shared that they actively advocated for better working conditions. Several participants shared that they had either individually or collectively urged leadership for safer workplaces.

### Suggestions for Workplace Safety Improvements

*They don’t really appreciate us, but I know [myself and] fellow colleagues, we are like heroes, and I feel like we should at least get hazardous pay, but I don’t know if that is ever going to happen. - Maya*

Focus group participants shared several suggestions at the transit authority and government level for COVID-19 workplace safety improvements. At the transit level, participants stated the need for improvements in cleaning practices, like updated air-filtration systems and more in-house cleaning staff. Participants also stated that there was a need for more consistent, streamlined communication and enforcement of COVID-19 safety protocols by leadership, such as for contact tracing and mask enforcement. Participants additionally shared the need for paid time off to get tested, vaccinated, and quarantine at home if sick from COVID-19.

*I feel like the government should support transit workers more, especially in this period of time because things are not easy for us. - Shauna*

At the government level, several participants expressed the need for increased government funding and resources directed to transit agencies for COVID-19 safety improvements. One participant suggested the scaling back of funding for aesthetic improvements at transit sites in order to reallocate those funds to protecting the health and safety of transit workers. Another participant suggested that the government needed to financially incentivize transit agencies to take action towards improving transit worker safety during the pandemic. Several participants stated the need for hazard pay given the high-risk job that transit workers engage in. Relatedly, participants shared that they wanted the government and public to view them as essential workers, thus providing them with the same benefits and resources as other occupations deemed essential.

### Role of Racism

*I think, you know, the darker you are, the harder it’s going to be across the board. It’s just no denying it. I definitely do feel as though if I was white, it’d be somewhat different as far as the response. - Aliyah*

A couple of participants did not believe that racism played a role in their experiences with COVID-19 as transit workers. As one participant stated, “I think what was going to happen from COVID, whatever my race was, it was going to be the same result.” However, the majority of participants believed that racism due to their marginalized racial identity factored into the lack of basic COVID-19 protections that they received as transit workers. In the exit survey, 90% of participants indicated that they were “concerned” or “very concerned” about racism impacting their health and safety at work, and 40% reported experiencing increased levels of racism during the pandemic. One participant noted that the demographics of the transit workforce had shifted over time from predominately white men to predominantly workers of color and women. This shift had led to differential power dynamics between transit drivers, who were mostly Black and Latinx, and transit leadership, who remained largely white. Another participant stated that they believed that hazard pay, safe working conditions, PPE, and additional funding for health and safety were not provided to the transit workforce because of their race.

*They’re going to put more funding out there [in higher income white neighborhoods] to clean the trains and the buses than they’re going to do in the inner city. Because you know what they probably say, it’s going to be dirty by the end of the day anyway. – Jamil*

At the structural level, participants mentioned the differential health and safety standards at transit stations in predominantly white, higher income neighborhoods compared to predominantly lower income Black and Brown neighborhoods. These participants believed that the intentional disinvestment in inner city transit sites was by design and due to racism.

## Discussion

Experiences both at home and in the workplace shaped behavioral intentions of Black public transit workers towards COVID-19 vaccination. Factors including a perceived rushed vaccine development timeline, inconsistent public health messaging from transit leadership and government agencies, and a lack of data on long-term effects of the COVID-19 vaccine led to uncertainty among study participants. However, being personally affected by the virus and enabling conditions in the workplace arose as top influencers of COVID-19 vaccine uptake.

Personal experience with COVID-19 loss served not only as catalyst for vaccination, but also uptake of other health protective behaviors like use of PPE and consistent hand hygiene. This finding aligns with previously published studies highlighting personal experiences as strongly influencing COVID-19 vaccine acceptance among Black communities in the USA and around the world [27, 28]. Studies have also found that inconsistent messaging from sources like the media, healthcare providers, and government agencies has led to uncertainty around COVID-19 preventative behaviors like vaccination [29–33]. While respondents were more likely to listen to reliable sources of vaccine guidance from healthcare providers and state and local health departments, conflicting and incomplete messaging across various sources ushered in confusion on what protective measures to employ in the workplace. This led to feelings of lack of support from leadership and a loss of agency in protecting themselves in the early stages of the pandemic. Accurate and transparent communication proved to be necessary in engraining trust and improving the uptake of COVID-19 preventative behaviors like vaccination among transit workers.

The negative impact of systemic racism on workplace safety emerged as a major theme from the focus group discussions. Participants highlighted their racial identity as a perceived contributor to lack of support from their assigned transit stations. Participants repeatedly articulated feeling devalued by transit leadership, the government, and transit ridership due to their combined racial and occupational status. This perceived devaluation aligns with existing scholarship on disposability politics, intersecting forms of oppression, and racial health inequities [34–36]. The COVID-19 pandemic illuminated in our society whose lives mattered and whose lives were expendable, often falling down race and class lines. Black transit workers, like many other essential workers of color, were thrust into the frontline of the pandemic with inadequate protections and increased susceptibility to disease and death. With limited ability to social distance, limited access to PPE, poor ventilation, and unsanitary working conditions, the Black transit workers interviewed were left to fend for themselves by the predominately white decision-makers in which they figuratively and literally worked under. Leadership and employers in the public transportation sector should address these biases not just for the benefit of the workers, but also for transit ridership.

The vast majority of research participants indicated that the government and their employers failed to take appropriate actions to protect their health and safety in the workplace during the pandemic. From a federal perspective, a proposed solution is to allocate a higher proportion of government funding and resources towards health and safety improvements like enhanced ventilation and regular sanitation services at public transit sites [37]. One substantial opportunity for funding is the Bipartisan Infrastructure Law (Infrastructure Investment and Jobs Act), which was passed in November 2021 and will provide $89.9 billion in investments to fund public transit over the next five years [38]. It is imperative that such federal investments be distributed equitably to ensure that Black transit workers and transit sites in predominately Black and lower income areas have access to public transit improvements. Moreover, transit agencies should ensure that health communications targeted towards transit workers and ridership are more clearly articulated and consistently enforced around COVID-19 and future pandemic prevention strategies like mask usage. In addition, transit agencies should work closely with labor unions and state and local governments to develop policies surrounding paid sick leave for COVID-19 infection, paid time-off for COVID-19 vaccination and testing, and hazard pay or bonuses to transit workers [39]. Transit authorities and government should also consider provisions, such as enforcement of safety planning committees, to protect transit workers from the uptick in violence they have experienced since the start of the COVID-19 pandemic [40].

These findings should be interpreted in light of limitations regarding the number, size, and composition of focus groups; the study comprised 10 participants and three focus groups. These limitations were due to pragmatic constraints, including disproportionate responses to recruitment outreach by metropolitan area, difficulty accommodating the busy schedules of transit workers, and inability to conduct the focus groups in-person due to the ongoing COVID-19 pandemic. Despite these practical challenges, the collected data and analytical approach enabled us to achieve our research objectives and obtain data sufficiency [41]. Given that this study was exploratory in nature, focus groups of a smaller size were deemed appropriate and allowed for more lively and engaging discussion between participants and between facilitators and participants [41, 42]. Upon analyzing transcripts for the three focus groups, we found sufficient redundancy in codes across the three focus groups to meet the threshold of data sufficiency [41, 43]. The study also has limited generalizability because participants worked primarily within one city. However, these findings may be applicable to other large, metropolitan public transit systems within the USA.

Despite these limitations, this study provides a deeper understanding of the barriers to vaccination for Black Americans. Because transit workers uphold critical infrastructure that is essential for the function of many communities, it is crucial that their safety and well-being are better protected in times of crises. Our findings indicate that there are numerous opportunities to improve both vaccine uptake and working conditions for Black transit workers by transit agencies and government officials.

## Appendix

**Table 1:** Demographic characteristics of participants (*n*=10)

**Characteristics**	**Value**
Demographics	
Age (years), median (IQR)	48 (36,52)
Gender, *n* (%)	
Male	6 (60)
Female	4 (40)
Prefer not to say	0 (0)
US City, *n* (%)	
Atlanta	1 (10)
New York	9 (90)
Highest level of education completed, *n* (%)	
High school graduate	2 (20)
Some college, no degree	3 (30)
Four-year college or university degree	4 (40)
Postgraduate or professional degree	1 (10)
Prefer not to say	0 (0)
Household’s total annual income (US dollars), *n* (%)	
$50,000-$75,000	1 (10)
$75,000-$100,000	6 (60)
$100,00 or more	3 (30)
Prefer not to say	0 (0)
Employment status, *n* (%)	
Full-time	10 (100)
Part-time	0 (0)
Prefer not to say	0 (0)
Type of transit operator, *n* (%)	
Bus operator	2 (20)
Train operator	3 (30)
Other	5 (50)
COVID-19 related questions, *n* (%)	
Have you ever tested positive for COVID-19?	
Yes	6 (60)
No	4 (40)
Prefer not to say	0 (0)
Do you think your COVID-19 infection was caused by exposure at work?	
Yes	5 (83)
No	0 (0)
Not sure	1 (17)
Occupational health	
In your view, have your employer’s actions to protect your health and safety been appropriate?	
Yes	0 (0)
No	9 (90)
Prefer not to say	1 (10)
Have you experienced increased levels of racism during the COVID-19 pandemic?	
Yes	4 (40)
No	5 (50)
Prefer not to say	1 (10)
How concerned are you about racism impacting your health and safety at work?	
1. Not concerned	1 (10)
2.	0 (0)
3.	3 (30)
4. Very concerned	6 (60)
Were you provided with proper and sufficient personal protective equipment (e.g., masks, masks, face shields, gloves, N95) by your employer?	
Yes	5 (50)
No	5 (50)
Prefer not to say	0 (0)
Has your employer retaliated against you for raising a safety concern or taking an action to protect the health of you or your co-workers?	
Yes	4 (40)
No	6 (60)
Prefer not to say	0 (0)
COVID-19 vaccine	
Have you received at least one dose of the COVID-19 vaccine?	
Yes	4 (40)
No	6 (60)
Prefer not to say	0 (0)
Vaccine communications	
When seeking information on the COVID-19 vaccine how likely are you to turn to the following for information?	
A doctor, nurse, or other health care provider	
1. Very likely	6 (60)
2.	1 (10)
3.	0 (0)
4. Very unlikely	3 (30)
Family or friends	
1. Very likely	3 (30)
2.	1 (10)
3.	3 (30)
4. Very unlikely	3 (30)
The Centers for Disease Control and Prevention, also known as the CDC	
1. Very likely	2 (20)
2.	3 (30)
3.	2 (20)
4. Very unlikely	3 (30)
Your state or local public health department	
1. Very likely	1 (10)
2.	5 (50)
3.	1 (10)
4. Very unlikely	3 (30)
A pharmacist	
1. Very likely	4 (40)
2.	2 (20)
3.	1 (10)
4. Very unlikely	3 (30)
A religious leader such as minister, pastor, priest, or rabbi	
1. Very likely	2 (20)
2.	1 (10)
3.	1 (10)
4. Very unlikely	6 (60)

## References

[1] Rogers TN, Rogers CR, VanSant-Webb E, Gu LY, Yan B, Qeadan F (2020). Racial disparities in COVID-19 mortality among essential workers in the United States. World Med Health Policy.

[2] Wolfe R, Harknett K, Schneider D. Inequalities at work and the toll of COVID-19. Health Aff Health Policy Brief. 2021;4

[3] Yearby R, Mohapatra S (2020). Law, structural racism, and the COVID-19 pandemic. J Law Biosci.

[4] Gershon RR, Merdjanoff AA, Meltzer GY, Piltch-Loeb R, Rosen J, Faiha CIH, Nwankwo EM, Medina P, Vlahov D, Sherman MF (2021). Impact of occupational exposure to COVID-19 on the physical and mental health of an essential workgroup: New York City transit workers. J Emerg Manag.

[5] Heinzerling A, Vergara XP, Gebreegziabher E (2022). COVID-19 outbreaks and mortality among public transportation workers — California, January 2020–May 2022. Morb Mortal Wkly Rep.

[6] Nguyen LH, Joshi AD, Drew DA, Merino J, Ma W, Lo CH, Kwon S, Wang K, Graham MS, Polidori L, Menni C (2021). Racial and ethnic differences in COVID-19 vaccine hesitancy and uptake.

[7] Bogart LM, Dong L, Gandhi P, Ryan S, Smith TL, Klein DJ, Fuller LA, Ojikutu BO (2021). What contributes to COVID-19 vaccine hesitancy in Black communities, and how can it be addressed?.

[8] MacDonald NE (2015). Vaccine hesitancy: definition, scope and determinants. Vaccine.

[9] Savoia E, Piltch-Loeb R, Goldberg B, Miller-Idriss C, Hughes B, Montrond A, Kayyem J, Testa MA (2021). Predictors of COVID-19 vaccine hesitancy: socio-demographics, co-morbidity, and past experience of racial discrimination. Vaccines.

[10] Willis DE, Andersen JA, Bryant-Moore K, Selig JP, Long CR, Felix HC, Curran GM, McElfish PA (2021). COVID-19 vaccine hesitancy: race/ethnicity, trust, and fear. Clin Transl Sci.

[11] Padamsee TJ, Bond RM, Dixon GN, Hovick SR, Na K, Nisbet EC, Wegener DT, Garrett RK (2022). Changes in COVID-19 vaccine hesitancy among Black and White individuals in the US. JAMA Netw Open.

[12] Smith AC, Woerner J, Perera R, Haeny AM, Cox JM (2022). An investigation of associations between race, ethnicity, and past experiences of discrimination with medical mistrust and COVID-19 protective strategies. J Racial Ethn Health Disparities.

[13] Yancy CW. COVID-19 and african americans. Jama. 2020;323(19):1891–92. 10.1001/jama.2020.6548 32293639

[14] Centers for Disease Control and Prevention. (Sept. 15 (2022). Risk for COVID-19 infection, hospitalization, and death by race/ethnicity.

[15] Garnier R, Benetka JR, Kraemer J, Bansal S (2021). Socioeconomic disparities in social distancing during the COVID-19 pandemic in the United States: observational study. J Med Internet Res.

[16] Poteat T, Millett GA, Nelson LE, Beyrer C (2020). Understanding COVID-19 risks and vulnerabilities among black communities in America: the lethal force of syndemics. Ann Epidemiol.

[17] Reyes MV (2020). The disproportional impact of COVID-19 on African Americans. Health Hum Rights.

[18] Brown H, Fremstad S, Rho HJ (2020). Racial inequality among workers in frontline industries: Black workers are overrepresented and undercompensated.

[19] Metropolitan Transportation Authority (2020). Diversity Committee Meeting.

[20] Rubinstein D (2020). Subway and bus workers are bearing a disproportionate coronavirus death toll.

[21] McClure ES, Vasudevan P, Bailey Z, Patel S, Robinson WR (2020). Racial capitalism within public health—how occupational settings drive COVID-19 disparities. Am J Epidemiol.

[22] Pirtle WNL (2020). Racial capitalism: a fundamental cause of novel coronavirus (COVID-19) pandemic inequities in the United States. Health Educ Behav.

[23] Foner PS, Kelley RDG. Organized Labor and the Black Worker, 1619–1981. Haymarket Books; 2018.

[24] Trotter JW (2019). Workers on arrival: Black labor in the making of America.

[25] Fishbein M, Ajzen I (2011). Predicting and changing behavior: the reasoned action approach.

[26] Brewer NT, Chapman GB, Rothman AJ, Leask J, Kempe A (2017). Increasing vaccination: putting psychological science into action. Psychol Sci Public Interest.

[27] Lazarus JV, Wyka K, White TM, Picchio CA, Rabin K, Ratzan SC, Parsons Leigh J, Hu J, El-Mohandes A (2022). Revisiting COVID-19 vaccine hesitancy around the world using data from 23 countries in 2021. Nat Commun.

[28] Momplaisir F, Haynes N, Nkwihoreze H, Nelson M, Werner RM, Jemmott J (2021). Understanding drivers of coronavirus disease 2019 vaccine hesitancy among blacks. Clin Infect Dis.

[29] Hornik R, Kikut A, Jesch E, Woko C, Siegel L, Kim K (2021). Association of COVID-19 misinformation with face mask wearing and social distancing in a nationally representative US sample. Health Commun.

[30] Lee JJ, Kang KA, Wang MP, Zhao SZ, Wong JYH, O'Connor S, Yang SC, Shin S (2020). Associations between COVID-19 misinformation exposure and belief with COVID-19 knowledge and preventive behaviors: cross-sectional online study. J Med Internet Res.

[31] Mheidly N, Fares J (2020). Leveraging media and health communication strategies to overcome the COVID-19 infodemic. J Public Health Policy.

[32] Motta M, Sylvester S, Callaghan T, Lunz-Trujillo K. Encouraging COVID-19 vaccine uptake through effective health communication. Front Polit Sci. 2021;3

[33] Nagler RH, Vogel RI, Gollust SE, Rothman AJ, Fowler EF, Yzer MC (2020). Public perceptions of conflicting information surrounding COVID-19: results from a nationally representative survey of US adults. PloS One.

[34] Bowleg L (2020). We’re not all in this together: On COVID-19, intersectionality, and structural inequality. Am J Public Health.

[35] Jones CP (2014). Systems of power, axes of inequity: parallels, intersections, braiding the strands. Med Care.

[36] Powell C (2021). Color of COVID and gender of COVID: essential workers, not disposable people. Yale JL Fem.

[37] Kuehn BM (2022). Bus and urban transit workers have highest COVID-19 risk. JAMA.

[38] The White House (2022). President Biden’s Bipartisan Infrastructure Law.

[39] Kinder M (2021). With federal aid on the way, it’s time for state and local governments to boost pay for frontline essential workers.

[40] Mueller E (2022). ‘Ticking time-bomb’: lag in protections for transit workers could hamper hiring and system upgrades.

[41] Suri H (2011). Purposeful sampling in qualitative research synthesis. Qual Res J.

[42] Tang KC, Davis A (1995). Critical factors in the determination of focus group size. Fam Pract.

[43] Krueger RA (2014). Focus groups: a practical guide for applied research.

